# Fitz-Hugh-Curtis syndrome associated with tuberculous salpingitis and peritonitis: a case presentation and review of literature

**DOI:** 10.1186/s12876-018-0768-0

**Published:** 2018-03-20

**Authors:** Laura Coremans, Frederik de Clerck

**Affiliations:** 1 Ghent University Hospital/AZ Sint-Lucas Ziekenhuis, Groene Briel 1, 9000 Ghent, Belgium; 2 Department of Hepatology and Gastroenterology, AZ Sint-Lucas, Groene Briel 1, 9000 Ghent, Belgium

**Keywords:** Fitz-Hugh-Curtis syndrome, Peritoneal tuberculosis, Tuberculous salpingitis, Ascites

## Abstract

**Background:**

Fitz-Hugh-Curtis syndrome or acute perihepatitis is considered a rare complication of pelvic inflammatory disease, mostly associated with chlamydial or gonococcal salpingitis.

Peritoneal tuberculosis is a rare site of extra-pulmonary infection caused by *Mycobacterium tuberculosis*. Infection usually occurs after reactivation of latent tuberculous foci in the peritoneum and more seldom after contiguous spread from tuberculous salpingitis.

**Case presentation:**

We describe a case of a 21-year old female of Somalian origin diagnosed with Fitz-Hugh Curtis syndrome associated with tuberculous salpingitis and peritonitis, presenting with new onset ascites.

Acid fast stained smear and polymerase chain reaction for *Mycobacterium tuberculosis* on ascitic fluid, endocervical culture and tuberculin skin test were all negative. Eventually, the diagnosis was made laparoscopically, showing multiple peritoneal white nodules and perihepatic “violin string” fibrinous strands.

**Conclusions:**

To our knowledge, this is the first case where Fitz-Hugh-Curtis syndrome is associated with both peritoneal and genital tuberculosis and where ascites was the primary clinical finding. Female genital tuberculosis has only rarely been associated with Fitz-Hugh-Curtis syndrome and all cases presented with chronic abdominal pain and/or infertility. Ascites and peritoneal involvement was not present in any case. Moreover, most patients with Fitz-Hugh-Curtis syndrome show no evidence of generalized intra-abdominal infection and only occasionally have concomitant ascites.

## Background

Peritoneal tuberculosis is a rare site of extra-pulmonary infection caused by *Mycobacterium tuberculosis* (TB). Infection usually occurs after reactivation of latent tuberculous foci in the peritoneum, after hematogenous spread from primary pulmonary TB [1, 2]. Less frequently transmural translocation from an infected small intestine or contiguous spread from tuberculous salpingitis occurs.

Fitz-Hugh-Curtis syndrome is defined as acute perihepatitis with typical “violin-string” adhesions between the liver and the anterior abdominal wall or the diaphragm, associated with pelvic inflammatory disease [3]. In the majority of cases it is associated with chlamydial or gonococcal salpingitis.

We present a case of Fitz-Hugh Curtis syndrome associated with tuberculous salpingitis and peritonitis, presenting with new onset ascites.

## Case presentation

A 21-year old female presented on the gastroenterology outpatient clinic with high-grade fever for 3 days and epigastric pain for 3 weeks. She was of Somalian origin and was living in Belgium since 2 years.

Her medical history consisted of malaria in her childhood and one month prior she gave birth to her first child. The labor was induced with secondary cesarean section because of general discomfort, raised inflammatory laboratory markers and raised liver function tests of unknown cause.

Clinical examination revealed abdominal distention and tenderness in the epigastric region without muscular defense. She denied having vaginal purulent discharge and lower abdominal pain. Her vital signs demonstrated a blood pressure of 100/73 mmHg, a heart rate of 124 bpm and a body temperature of 38 °C.

Laboratory studies revealed raised inflammatory markers (total WBC count of 11,700/mm^3^, CRP level of 170 mg/l), mild microcytic anemia (Hb 10.2 g/dl, MCV 76 fL), significant thrombocytosis (953,000 /mm^3^) and mildly raised liver function tests (AST 23 U/L, ALT 51 U/L, alkaline phosphatase 279 U/l, gamma-glutamyl transferase 74 U/l). Bilirubin, albumin and prothrombin time were normal.

Viral hepatitis, human immunodeficiency virus (HIV) and Malaria were excluded.

**Fig. 1:**
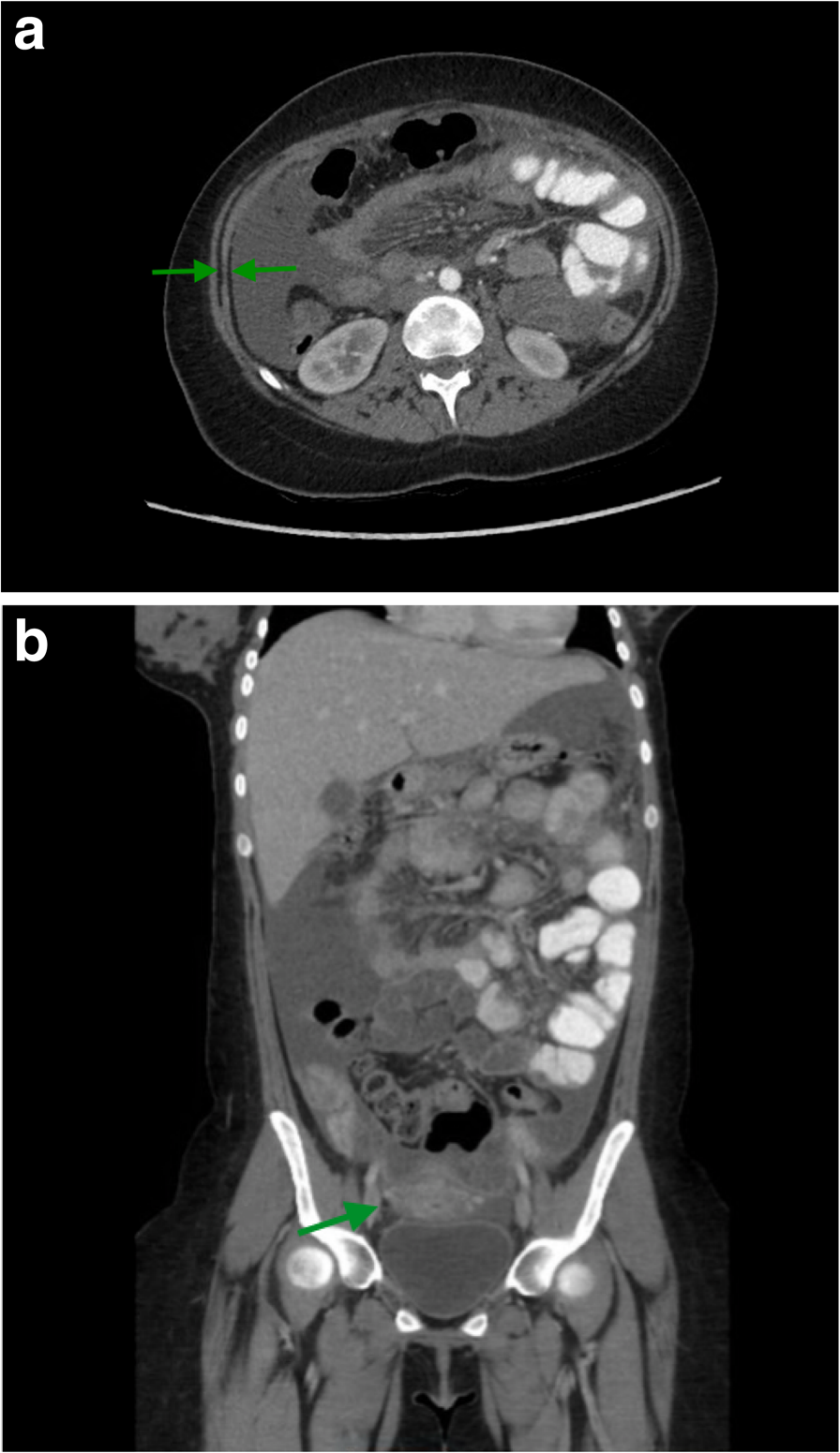
**a** and **b**. Contrast-enhanced CT images of the abdomen showing peritoneal thickening and hypervascular adnexes with a small para-uterine abcedation on the right side

Abdominal ultrasound confirmed the presence of ascites in the small pelvis and around the liver, with normal liver size and parenchyma. Subsequent abdominal computed tomography (CT) revealed peritoneal thickening and hypervascular adnexes with a small para-uterine abcedation on the right side, suggestive for pelvic inflammatory disease (PID) (Fig. 1). There were no enlarged lymph nodes and the liver veins were patent.

Diagnostic abdominal paracentesis with evacuation of purulent fluid was performed. Ascitic fluid analysis showed a serum-ascites albumin gradient less than 1.1 g/dL with cytology showing a predominance of lymphocytic cells.

Acid fast stained smear (Ziehl-Neelsen staining) and polymerase chain reaction (PCR) for *M. tuberculosis* on ascitic fluid were negative.

Tuberculin skin test was negative and there were no signs suggestive of (previous) tuberculosis on chest radiography.

Gynecological evaluation revealed purulent cervical discharge, cervical motion tenderness and adnexal tenderness. Endocervical culture was negative for Neisseria gonorrhoeae, Chlamydia trachomatis and Trichomonas vaginalis. Acid fast stained smear was also negative.

**Fig. 2:**
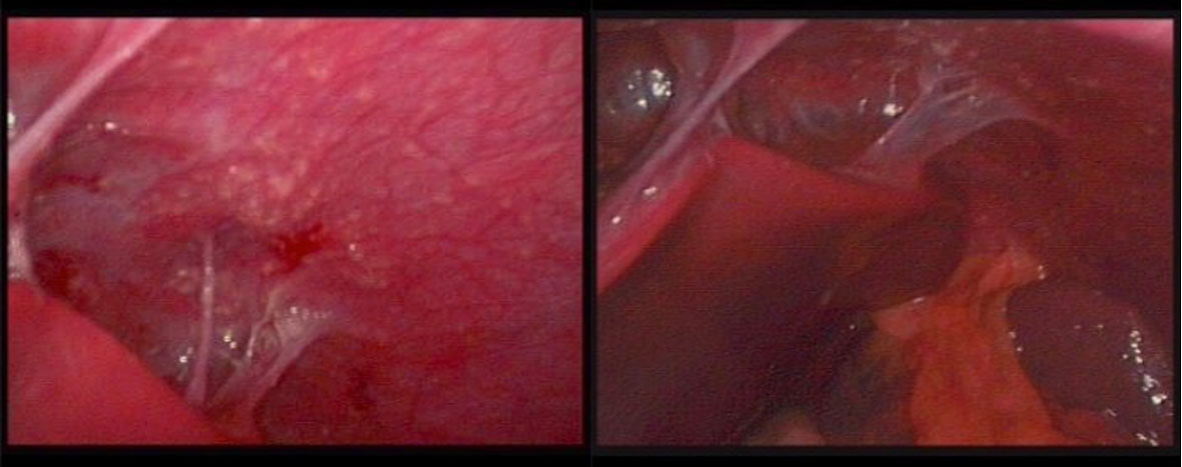
Intraoperative laparoscopic photo showing multiple peritoneal white nodules (left) and perihepatic “violin string” fibrinous strands (right)

Subsequently a diagnostic laparoscopy was performed, showing multiple peritoneal white nodules and perihepatic “violin string” fibrinous strands (Fig. 2). These findings were very suggestive for peritoneal tuberculosis with Fitz-Hugh Curtis syndrome.

Acid fast stained smear and PCR for *M. Tuberculosis* on peritoneal biopsies were both negative.

Since the perioperative findings were very suggestive, the patient was started on quadruple anti-tuberculous therapy, consisting of isoniazid, rifampin, pyrazinamide and ethambutol. Rapid clinical improvement and regression of the inflammatory markers and ascites was observed.

The pathology report of the peritoneal biopsies eventually confirmed peritonitis with granulomas.

Four weeks after starting anti-tuberculous therapy direct cultures for *M. Tuberculosis* on peritoneal biopsies and endocervical swab were reported as positive. Ascites cultures remained negative.

## Discussion and conclusions

To our knowledge, this is the first case where Fitz-Hugh-Curtis syndrome is associated with both peritoneal and genital tuberculosis and where ascites was the primary clinical finding. Sharma et al. already described 3 cases of Fitz-Hugh-Curtis syndrome associated with female genital tuberculosis, however they all presented with chronic abdominal pain and/or infertility [4]. Ascites and peritoneal involvement was not present in any case.

Fitz-Hugh-Curtis syndrome provides a diagnostic challenge as it can mimic many other diseases (most often acute cholecystitis). Clinical presentation includes sharp pain in the upper right quadrant, fever and in most cases, but not always, signs of salpingitis [5]. Most patients show no evidence of generalized intra-abdominal infection and only occasionally have concomitant ascites [3, 5].

Contrast-enhanced abdominal computed tomography usually shows linear contrast enhancement of the liver capsule [6]. This was not present in our case.

Symptoms and signs, physical examination and laboratory findings of peritoneal tuberculosis are nonspecific. Symptoms have an insidious onset and include ascites, diffuse abdominal pain, low-grade fever and weight loss, developing over a period of several weeks to months [7]. Lab abnormalities may include mild to moderate anemia, peripheral -usually lymphocytic- leukocytosis, increased alkaline phosphatase or transaminases and hypoalbuminemia [7].

Abdominal ultrasound may show typical fine mobile strands [8]. Computed tomography findings include peritoneal thickening, omental cake and enlarged mesenteric lymph nodes. Computed tomography findings include peritoneal thickening, omental cake and enlarged mesenteric lymph nodes.

Ascitic fluid analysis typically shows a serum-ascites albumin gradient (SAAG) less than 1.1 g/dL with protein level of more than 2.5 to 3 g/dL [9]. Cytology typically shows a predominance of lymphocytic cells.

The differential diagnosis of ascites with lymphocytic predominance and SAAG of less than 1.1 g/dL includes peritoneal carcinomatosis, nephrotic syndrome, pancreatitis and peritoneal tuberculosis [10]. The main advantage of calculating the SAAG is its specificity for ascites caused by portal hypertension [9]. A SAAG of more than 1.1 g/dL indicates portal hypertension with an accuracy of 97% [10].

Acid fast stained smear on specimens collected from sites of suspected extra-pulmonary TB has low sensitivity (less than 5% on peritoneal fluid). However because false-positive results are unlikely, recent guidelines recommend performing acid fast stained smear on ascitic fluid and peritoneal biopsies, under the condition that a negative result is not used to exclude peritoneal tuberculosis [11].

Gene amplification tests such as PCR to detect *M. tuberculosis* on ascitic fluid and peritoneal biopsies are another rapid and non-invasive test [11]. However low sensitivity has been reported in smear-negative patients (48%) [9].

Tuberculin skin testing is mostly used as a screening tool for latent tuberculosis, given its low sensitivity and low positive predictive value [9].

Elevated serum CA-125 levels (> 35 U/mL) and adenosine deaminase activity (ADA) of ascitic fluid (> 30 U/l) have been proposed as easy, non-invasive tests for peritoneal tuberculosis with high sensitivity and specificity (respectively 83% and 50% for CA-125 and 93% and 94% for ADA) [12, 13].Therefore measuring ADA levels is recommended in the diagnostic work-up for peritoneal tuberculosis [11].

Since CA-125 is a tumor marker associated with ovarian cancer, ovarian malignancy should be excluded before making the diagnosis of peritoneal tuberculosis in case of elevated CA-125 values, especially because the clinical manifestations may show high resemblance.

Some studies also suggest a role for serum CA-125 levels as a follow-up marker in monitoring the response to anti-tuberculous therapy [14, 15].

For a definite diagnosis of peritoneal tuberculosis, microbiological and/or histological confirmation is needed [16]. To date, direct culture of *M. tuberculosis* on ascitic fluid or peritoneal biopsies is the gold standard for diagnosis of abdominal tuberculosis, with a reported sensitivity of 45 to 69% [11]. However it can take up to 6 weeks before cultures become positive.

This means that in the majority of cases, as well as in the case presented here, diagnostic laparoscopy and direct visualization of the peritoneum is needed in the diagnostic process [16, 17]. Therefore a high index of clinical suspicion is needed to avoid delay in treatment initiation and risking increased mortality [9].

In most developed countries, laparoscopy is easily accessible, appears to be relatively safe with a reported complication rate of less than 3% and has a high diagnostic sensitivity (93%) and specificity (98%) [9]. In the case described herein laparoscopy also allowed for direct visualization and biopsy of the para-uterine abcedation described on abdominal CT.

In regions where laparoscopy is less easily accessible, percutaneous ultrasound- or CT-guided biopsy of the peritoneum or mesenteric lymph nodes can be considered as safe alternative with low incidence of complications [18, 19].

However in the presence of large amounts of ascites, ultrasound-guided biopsy is less appropriate as hemostasis during biopsy through local pressure with the transducer is difficult to achieve [20]. In our patient no easily accessible, pathologically enlarged lymph nodes were visualized.

Typical macroscopic findings include multiple white nodules or tubercles, enlarged lymph nodes, “violin string” fibrinous strands and omental thickening. Peritoneal carcinomatosis, sarcoidosis and Crohn’s disease may mimic the laparoscopic findings of peritoneal tuberculosis [9]. Even in the absence of histological or bacteriological confirmation, the characteristic laparoscopic appearance is sufficient reason for initiating anti-tuberculous therapy. Similarly, in ocular tuberculosis the diagnosis is frequently presumed based on macroscopic findings (solitary tubercles, miliary choroidal tubercles, tuberculoma’s e.g) as ocular tissue for microscopic evaluation is difficult to obtain [21].

Microscopic examination of peritoneal and lymph node biopsies in peritoneal tuberculosis show caseating granulomas in up to 100% of patients, as was the case in our patient [16].

Treatment for tuberculous peritonitis is the same as for pulmonary disease, with an intensive phase of 2 months of isoniazid, rifampin, pyrazinamide, and ethambutol followed by a continuation phase of 4 months of isoniazid and rifampin [22, 23]. Response to treatment is best assessed clinically, with resolution of symptoms and ascites [24].

## Abbreviations

ADA: Adenosine deaminase activity
PCR: Polymerase chain reaction
PID: Pelvic inflammatory disease
SAAG: Serum-ascites albumin gradient
TB: Tuberculosis
WBC: White blood cells count
